# Silencing of lncRNA MIAT alleviates LPS-induced pneumonia via regulating miR-147a/NKAP/NF-κB axis

**DOI:** 10.18632/aging.202284

**Published:** 2020-12-09

**Authors:** Min Liu, Weixin Li, Fuxing Song, Ling Zhang, Xianjun Sun

**Affiliations:** 1 Department of Pediatrics, Jinan People’s Hospital Affiliated to Shandong First Medical University, Jinan, Shandong Province, China; 2 Department of Infectious Diseases, Jinan Hospital of Integrated Traditional Chinese and Western Medicine, Jinan, Shandong Province, China

**Keywords:** pneumonia, lncRNA MIAT, miR-147a, NKAP, LPS

## Abstract

Purpose: Pneumonia is a respiratory disease with an increasing incidence in recent years. More and more studies have revealed that lncRNAs can regulate the transcriptional expression of target genes at different stage. Herein, we aimed to explore the effect of lncRNA MIAT in LPS-induced pneumonia, and further illuminate the possible underlying mechanisms.

Method and results: Mice were intraperitoneally injected with LPS, and the lung inflammation was evaluated. Microarray showed lncRNA MIAT was up-regulated in LPS-induced pulmonary inflammation. And qRT-PCR and FISH assay indicated that MIAT was increased in mice with LPS injection. Functional analysis showed sh-MIAT inhibited LPS-induced inflammation response, inhibited apoptosis level and protected lung function. As well, si-MIAT removed the injury of LPS on mouse lung epithelial TC-1 cells, and inhibited the activation of NF-κB signaling. Furthermore, MIAT acted as a sponge of miR-147a, and miR-147a directly targeted NKAP. Functionally, AMO-147a or NKAP remitted the beneficial effects of si-MIAT on LPS-induced inflammation response of TC-1 cells.

Conclusion: Deletion of MIAT protected against LPS-induced lung inflammation via regulating miR-147a/NKAP, which might provide new insight for pneumonia treatment.

## INTRODUCTION

Pneumonia is a respiratory disease with an increasing incidence in recent years [1]. In particular, children are extremely prone to pneumonia, with an incidence of up to 30% in the peak season [2]. When the pathogenic microorganism invades the host, it can stimulate the immune system to produce inflammatory factors, which accumulate locally [3]. On the other hand, the descending infection department caused respiratory tract infection, and the clinical manifestations were not bronchitis, pneumonia and so on. In severe cases, it can accumulate in the brain, heart, liver and kidney, followed by encephalitis, myocarditis and hepatitis and other complications [4]. However, at present, the pathogenesis of childhood pneumonia is still controversial and there are still difficulties in clinical treatment, so it is of great significance to explore the pathogenesis of childhood pneumonia at the molecular level.

More than 80% of the genes in the human genome are transcribed, yet less than 2% of the genes encode proteins [5]. In addition to tRNA and rRNA, these non-coding transcriptional sequences have a variety of regulatory RNA [6]. In the past 10 years, researchers have focused on the study of non-coding RNA. With the development of gene chip, high-throughput sequencing, RNA pull-down and other technologies, lncRNA has gradually entered people's attention and become a research hotspot at home and abroad [7]. At first, people thought that lncRNA is the "noise" of genome transcription [8]. However, recent studies have found that lncRNA is involved in transcriptional regulation, post-transcriptional regulation, genomic imprinting, epigenetics and so on [9]. LncRNA can also be used as a "bait" to combine with miRNA to play the "sponge" role of miRNA, thus regulating a series of pathophysiological processes [10]. It has been found that lncRNA and miRNA play an important role in the occurrence, development, treatment and prognosis of respiratory diseases [11].

Pneumonia is often accompanied by lung injury [12, 13], which turns into pulmonary edema and neutrophil accumulation [14, 15]. Zhang et al. [16] found that lncRNA FOXD3-AS1 was the most significantly upregulated lncRNAs in hyperoxia-induced acute lung injury model. The team determined the luciferase activity of FOXD3-AS1 and miR-150 and found that FOXD3-AS1 could be used as a sponge for miR-150. In the model of lung injury, miR-150 has a cytoprotective effect, while FOXD3-AS1 can promote cell death, which may be that FOXD3-AS1, as the "sponge" of miR-150, limits the cytoprotective effect of miR-150, thus exaggerating the cell death induced by hyperoxia. In addition, Wu et al. [17] found that lncRNA H19 inhibits lipopolysaccharide-induced injury by inhibiting the expression of miR-181a and indirectly increasing the expression of Runx2, the downstream target of miR-181a. At the same time, the overexpression of Runx2 is related to the activation of JNK and Notch signal pathways. This study suggests that H 19 may be involved in acute lung injury by regulating JNK and Notch signal pathways through its sponge effect.

LncRNA MIAT (myocardial infarction associated transcript) is a kind of lncRNA which is mainly expressed in cardiomyocytes and kidney cells, and has the function of regulating protein synthesis [18, 19]. Linn Fagerberg et al. used microarray technology to analyze the transcriptome of human tissues and organs, and found that MIAT was highly expressed in bone marrow, indicating that bone marrow is one of the main sites of MIAT [20]. In addition, MIAT has been found to play an important role in the occurrence of many diseases, mainly related to the activation of inflammatory response [21, 22]. Therefore, we speculate that lncRNA MIAT may participate in the progression of pneumonia by regulating inflammatory response. Herein, we explored the effect of lncRNA MIAT in pneumonia, and further illuminate the possible underlying mechanisms.

## RESULTS

### The expression of lncRNA MIAT in LPS-induced pneumonia mice

**Figure 1:**
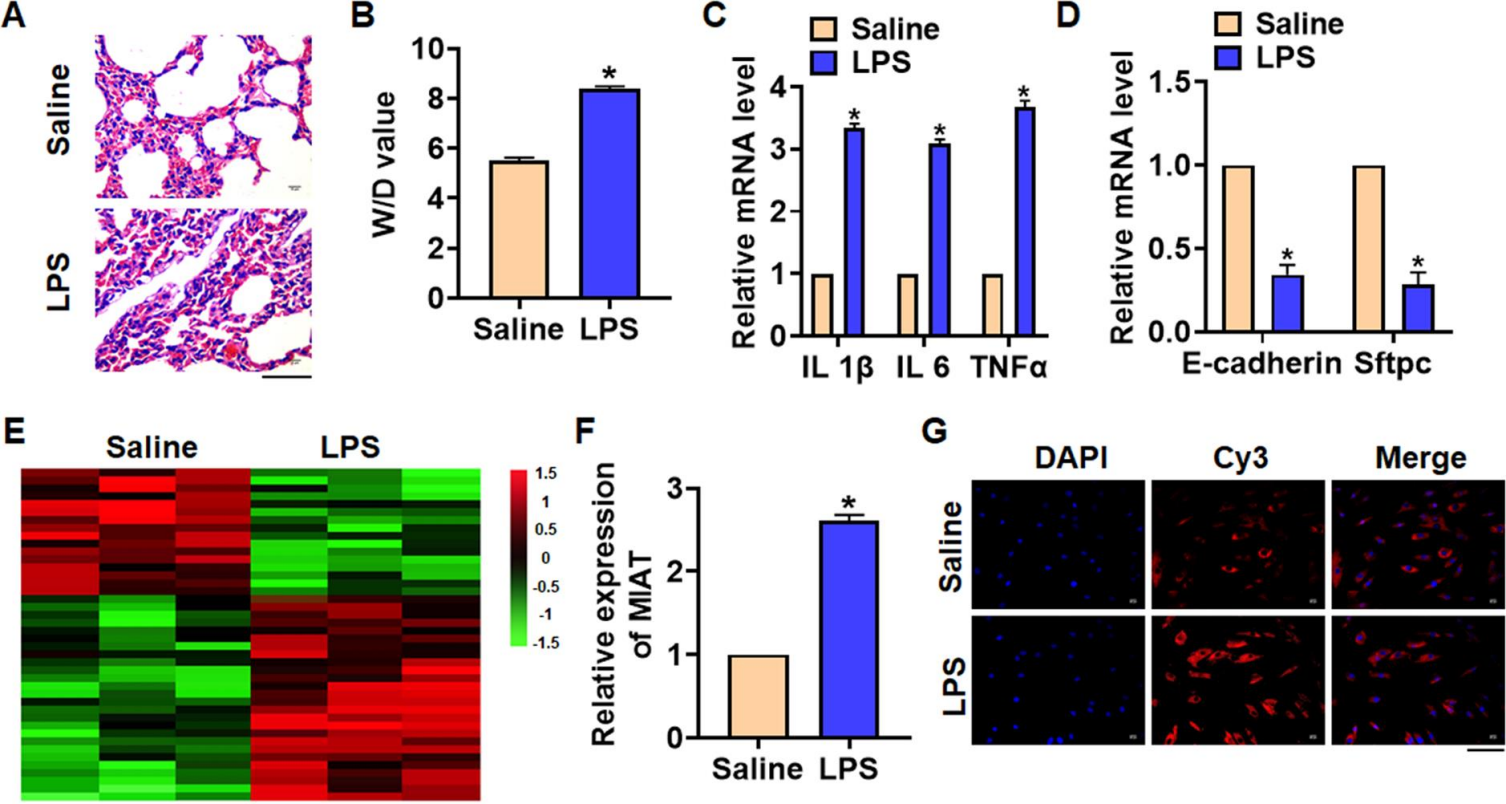
**The expression of lncRNA MIAT in LPS-induced pneumonia.** 10 μg LPS was intraperitoneally injected into mice to establish mouse model of pneumonia. (**A**) H&E staining for mice lung sections. Scale bar, 60 μm. (**B**) Wet dry mass ratio (W/D) of lungs was calculated. (**C**) The expression of inflammatory factors IL 1β, IL 6 and TNFα was detected by qRT-PCR. (**D**) The expression of epithelial markers E-cadherin and Sftpc was determined by qRT-PCR. (**E**) LncRNA expression profiles in mice with saline or LPS. (**F**) The expression of MIAT in saline and LPS injected lungs was detected by qRT-PCR. (**G**) FISH assay was used to determine the location and level of MIAT upon LPS treatment. Scale bar, 100 μm. Data are mean ± SD; *P < 0.05. All experiments were repeated three times.

We first established mouse model of pneumonia by intraperitoneally injecting LPS, and the inflammation of lungs was evaluated. LPS treatment significantly increased the infiltration of inflammatory cell and induced the pulmonary edema (Figure 1A). LPS injection increased wet dry mass ratio (W/D) of isolated lungs, indicating that LPS treatment induced pulmonary edema (Figure 1B). Then qRT-PCR also indicated that the expression of inflammatory factors IL 1β, IL 6 and TNFα was upregulated upon LPS stimulation (Figure 1C). Alveolar epithelial cells are involved in maintaining normal lung function, so we estimated alveolar epithelial cells function by examining epithelial markers expression. LPS administration reduced the mRNA expressions of E-cadherin and Sftpc (Figure 1D). Above data proved that the mouse pneumonia model was established successfully. Then, we performed Microarray analysis, and the data showed the differentially expressed lncRNAs in saline and LPS treatment of lung tissues, which showed an increase of MIAT in LPS-treated lung tissues (Figure 1E). Then qRT-PCR also indicated that MIAT was upregulated in LPS-treated lung tissues comparing with saline-treated lung tissues (Figure 1F). Then, FISH assay showed that MIAT expression, predominantly located in the cytoplasm, was dramatically raised in lung tissue of LPS stimulation (Figure 1G).

### Knockdown of MIAT alleviates LPS-induced inflammation and injury in mice

**Figure 2:**
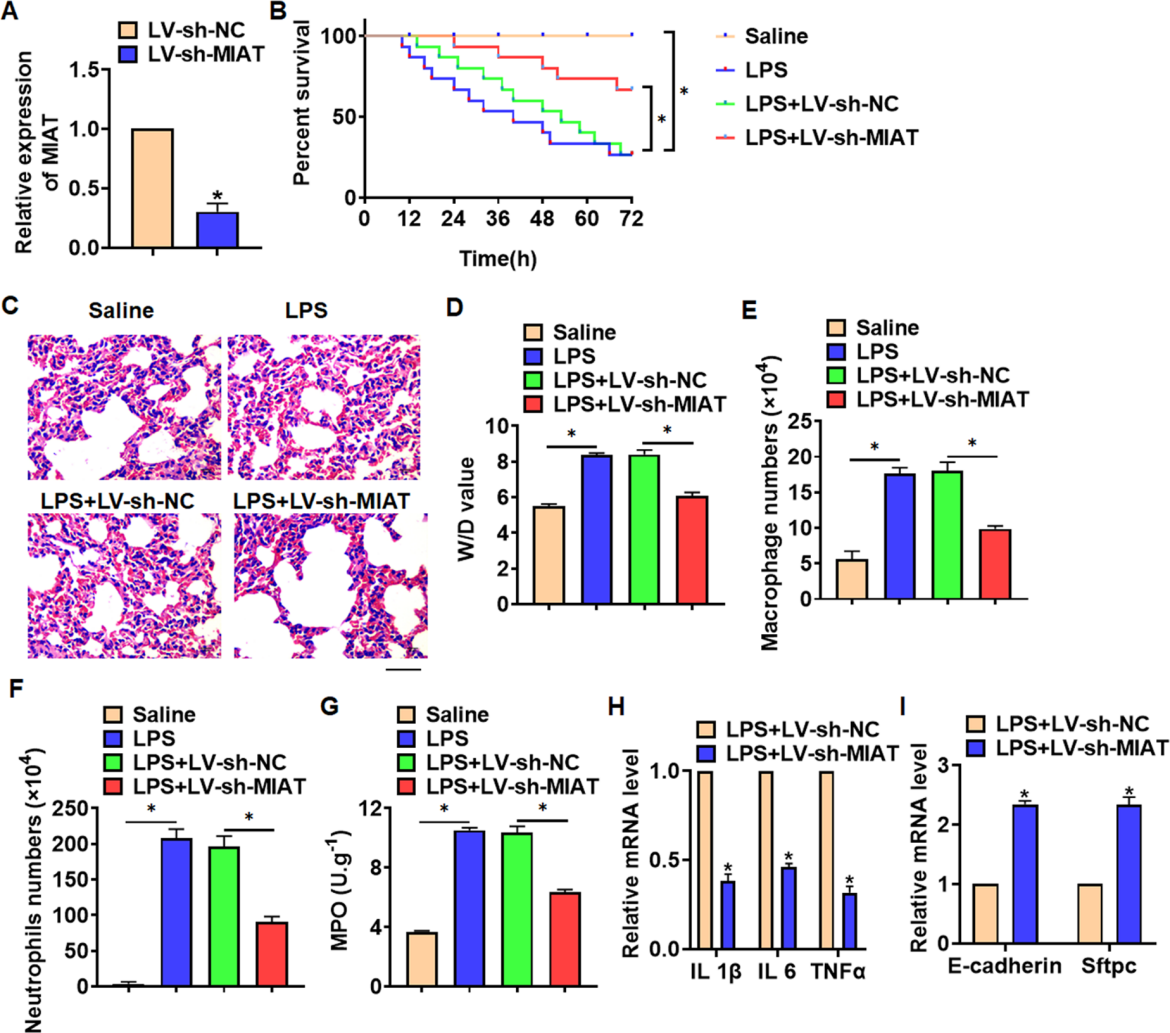
**Knockdown of MIAT alleviates LPS-induced inflammation and injury in mice.** LV-sh-MIAT or LV-sh-NC was intratracheally injected into mice. (**A**) The knockdown efficiency of sh-MIAT was determined by qRT-PCR. (**B**) Survival plots for mice in different groups. (**C**) H&E staining for lung sections in different groups. Scale bar, 60 μm. (**D**) Wet dry mass ratio (W/D) of lungs was calculated. (**E**, **F**) macrophages and neutrophils in alveolar lavage fluid was collected and calculated. (**G**) Myeloperoxidase (MPO) of lungs was examined. (**H**) qRT-PCR analysis for IL 1β, IL 6 and TNFα expression. (**I**) qRT-PCR analysis for E-cadherin and Sftpc expression. Data are mean ± SD; *P < 0.05. All experiments were repeated three times.

For further research, we constructed lentiviral plasmid for knockdown the expression of MIAT (LV-sh-MIAT, LV-sh-NC was indicated as a control group), and intratracheally injected into mice (Figure 2A). The survival curve showed that LPS significantly inhibited the survival rate of mice, while deletion of MIAT decreased the mice mortality compared with LPS group (Figure 2B). Histological examination showed that LPS caused structural damage of lung tissue in mice, accompanied by apparent inflammatory cell infiltration and alveolar hemorrhage. LV-sh-MIAT could significantly alleviate the above pathological changes induced by LPS (Figure 2C). LPS stimulation increased wet dry mass ratio (W/D) of isolated lungs, while LV-sh-MIAT injection decreased W/D, indicating that sh-MIAT inhibited pulmonary edema (Figure 2D). Then, we collected and calculated macrophages and neutrophils in alveolar lavage fluid LPS injection promoted the numbers of macrophages and neutrophils, while sh-MIAT inhibited the aggregation of macrophages and neutrophils (Figure 2E, 2F). As well, sh-MIAT inhibited the increase of myeloperoxidase (MPO) in LPS-treated lungs (Figure 2G). In addition, silencing of MIAT inhibited inflammatory factors expression (Figure 2H), and restored the level of E-cadherin and Sftpc comparing with LPS treatment group (Figure 2I).

### Deletion of MIAT attenuated LPS-induced inflammation and injury in cells

**Figure 3:**
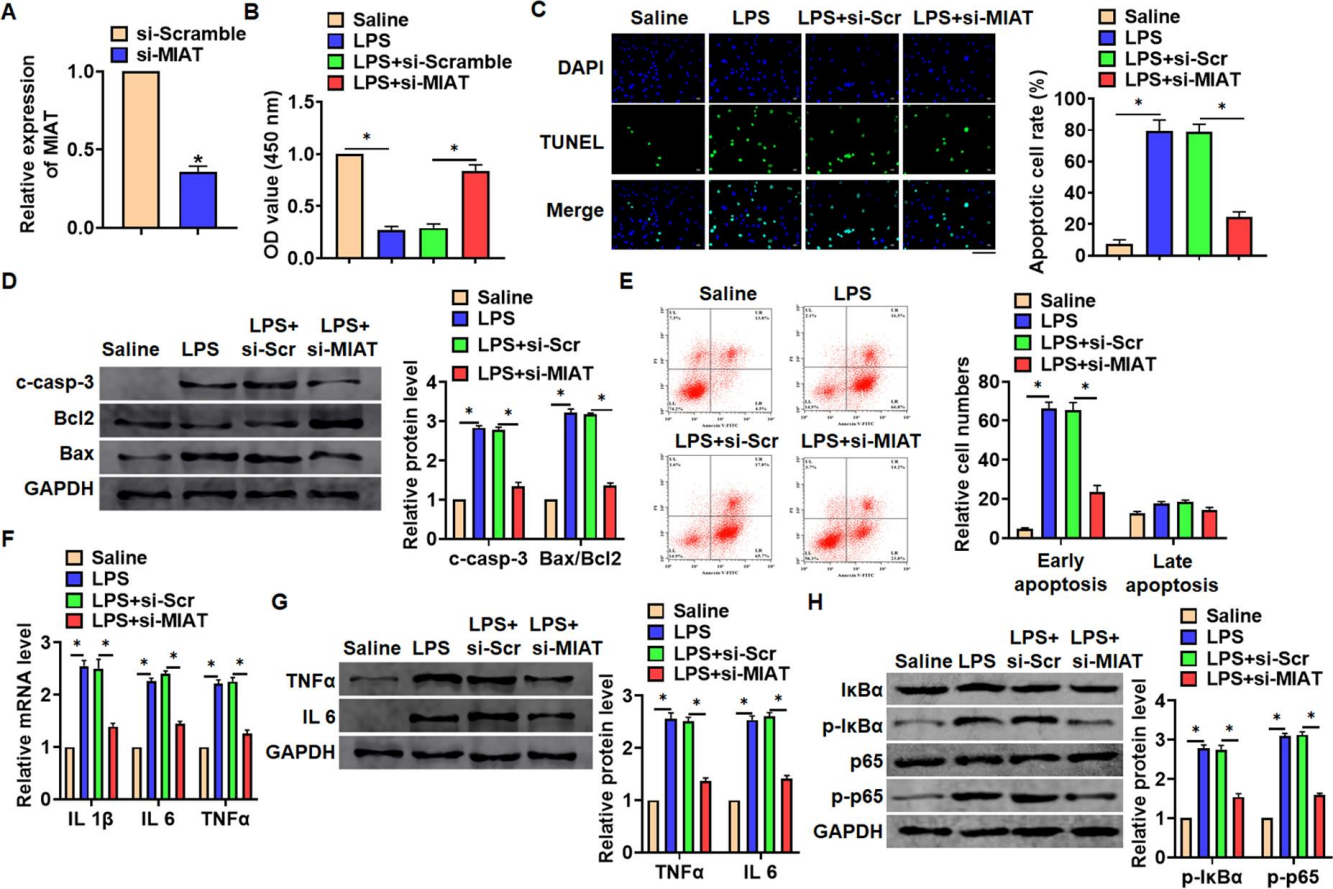
**Knockdown of MIAT attenuated LPS-induced inflammation and injury in cells.** siRNA of MIAT was transfected into TC-1 cells with LPS treatment (100 ng/mL). (**A**) The silencing efficiency of si-MIAT was detected by qRT-PCR. (**B**) MTT assay for cell viability of TC-1 cells. (**C**) Apoptosis cell numbers were tested by TUNLE staining. Scale bar, 100 μm. (**D**) Western blot for apoptosis related proteins (cleaved-caspase-3, Bax, Bcl2) in TC-1 cells. (**E**) Flow cytometry assay used to detect early and late apoptosis cell numbers. (**F**) qRT-PCR analysis for IL 1β, IL 6 and TNFα expression. (**G**) Western blot for TNFα and IL 6 expression. (**H**) Western blot for NF-κB signaling gene IκBα and p65. Data are mean ± SD; *P < 0.05. All experiments were repeated three times.

*In vitro*, we cultured TC-1 cells treated with LPS (100 ng/mL) to mimic *in vivo* LPS-induced pneumonia. And siRNA of MIAT was transfected into cells with LPS treatment (Figure 3A). MTT results showed that LPS treatment decreased cell viability, while si-MIAT recover cell viability and remitted the injury effects of LPS (Figure 3B). In addition, TUNEL analysis exhibited an increase of apoptotic cell numbers in LPS treated TC-1 cells, while si-MIAT decreased apoptotic cell numbers (Figure 3C). As well, LPS promoted the expression of cleaved-caspase-3 and Bax/Bcl2, which was reversed by si-MIAT transfection (Figure 3D). Flow cytometry assay showed that si-MIAT inhibited both early and late apoptotic cell numbers (Figure 3E). Moreover, LPS induced the expression of inflammation factors, while si-MIAT reduced inflammation factors level (Figure 3F, 3G). NF-κB signaling exerts an essential function in inflammation response. LPS treatment activated NF-κB signaling with increasing expression of p-IκBα and p-p65, but this effect was reversed by si-MIAT transfection (Figure 3H).

### MIAT interacted with miR-147a

**Figure 4:**
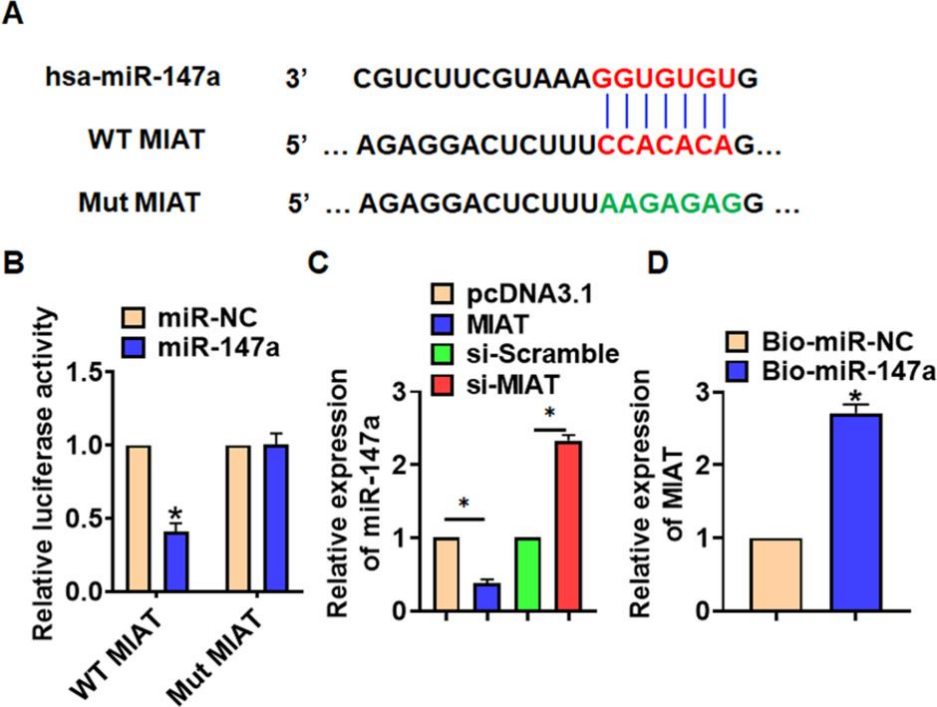
**MIAT acted as a sponge of miR-147a.** (**A**) MiRanda database showing the binding sites of miR-147a with MIAT, and the mutant sequence of MIAT. (**B**) Wild type and mutant MIAT was transfected into HEK293 cells with or without miR-147a, and luciferase assay was to evaluate the binding between miR-147a and MIAT. (**C**) TC-1 cells were transfected with MIAT plasmid or si-MIAT or its NC, the mRNA level of miR-147a was detected using qRT-PCR. (**D**) Biotinylated miR-147a or NC was transfected into H460 cells, and qRT-PCR was performed to detect the enrichment of MIAT. Data are mean ± SD; *P < 0.05. All experiments were repeated three times.

To explore the molecular mechanism of MIAT in LPS-induced pneumonia, we used miRanda database and found a potential binding between MIAT and miR-147a (Figure 4A). Then luciferase assay showed miR-147a inhibited activity of WT MIAT not mut MIAT in HEK293 cells (Figure 4B). And overexpression of MIAT inhibited miR-147a level, while silencing of MIAT promoted miR-147a level in TC-1 cells (Figure 4C). Further, endogenous MIAT was enriched in biotinylated miR-147a transfected TC-1 cells, which reveals a direct binding of MIAT with miR-147a (Figure 4D).

### MiR-147a inhibited NKAP expression

**Figure 5:**
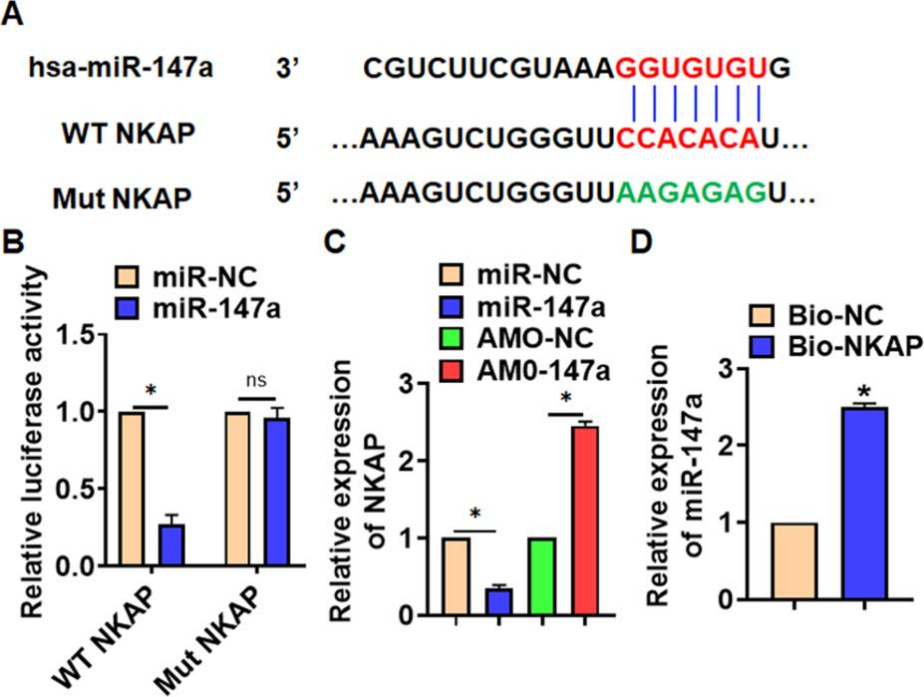
**NKAP was a directed target of miR-147a.** (**A**) The binding bases of miR-147a and NKAP from Targetscan. (**B**) Wild type and mutant NKAP was transfected into HEK293 cells with or without miR-147a, and luciferase assay was to evaluate the binding. TC-1 cells were transfected with miR-147a or AMO-147a, (**C**) the mRNA level of NKAP was detected. (**D**) RIP assay for the binding of miR-147a and NKAP in H460 cells. Data are mean ± SD; *P < 0.05. All experiments were repeated three times.

Through Targetscan we found bases pairing of miR-147a and NKAP (Figure 5A). Followed luciferase. analysis suggested miR-147a directly inhibited NKAP expression (Figure 5B). Furthermore, miR-147a suppressed NKAP mRNA and protein expression, but AMO-147a increased NKAP level in TC-1 cells (Figure 5C and 5D). RIP assay showed an enrichment of miR-147a in biotinylated NKAP cells.

### Si-MIAT alleviates LPS-induced inflammation and injury via miR-147a/NKAP axis

**Figure 6:**
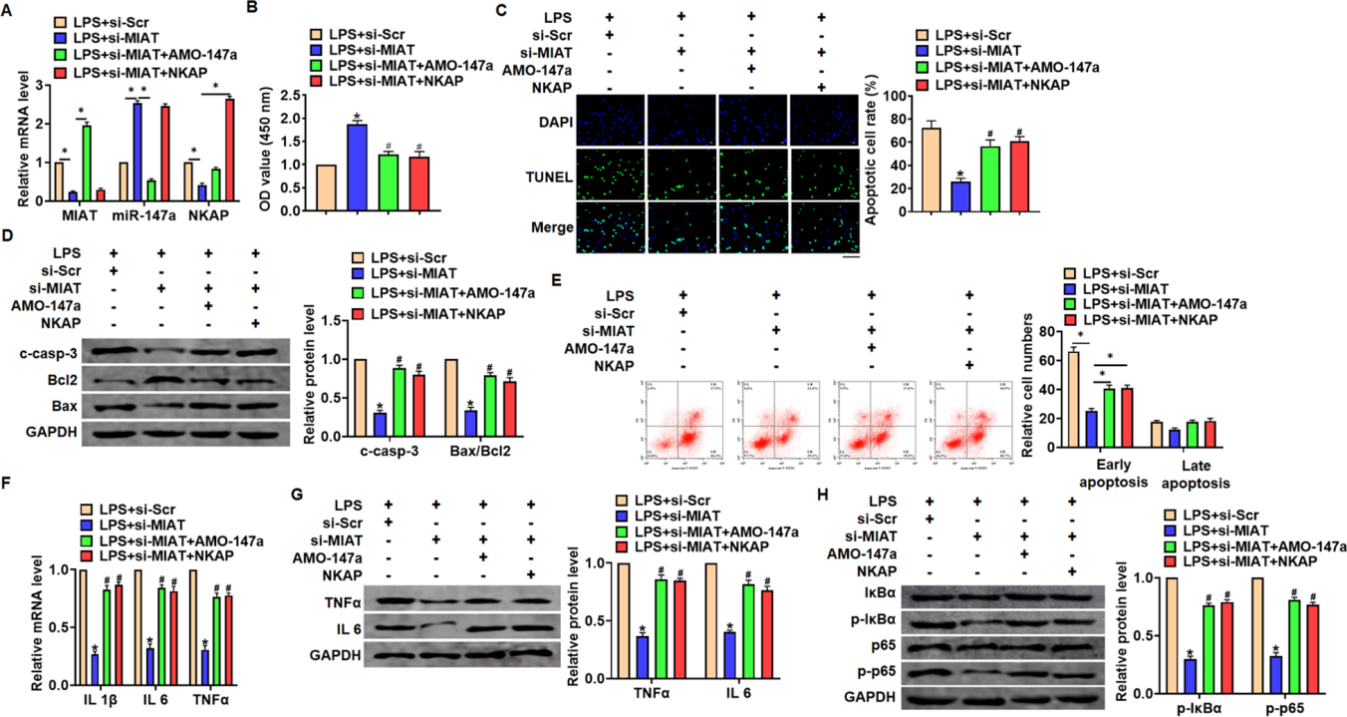
**Inhibition of MIAT alleviates LPS-induced inflammation and injury via miR-147a/NKAP axis in TC-1 cells.** Si-MIAT was transfected into TC-1 cells with AMO-147a or NKAP. (**A**) The transfection efficiency was detected using qRT-PCR. (**B**) MTT assay for cell viability of TC-1 (**C**) Apoptosis cell numbers were tested by TUNLE staining. Scale bar, 100 μm. (**D**) Western blot for cleaved-caspase-3, Bax, Bcl2 in TC-1 cells. (**E**) Flow cytometry assay used to detect early and late apoptosis cell numbers. (**F**) qRT-PCR analysis for IL 1β, IL 6 and TNFα expression. (**G**) Western blot for TNFα and IL 6 expression. (**H**) Western blot for IκBα and p65. Data are mean ± SD; *P < 0.05. Data are mean ± SD; *P < 0.05 vs LPS+si-Scramble, ^#^P< 0.05 vs LPS+si-MIAT. All experiments were repeated three times.

**Figure 7:**
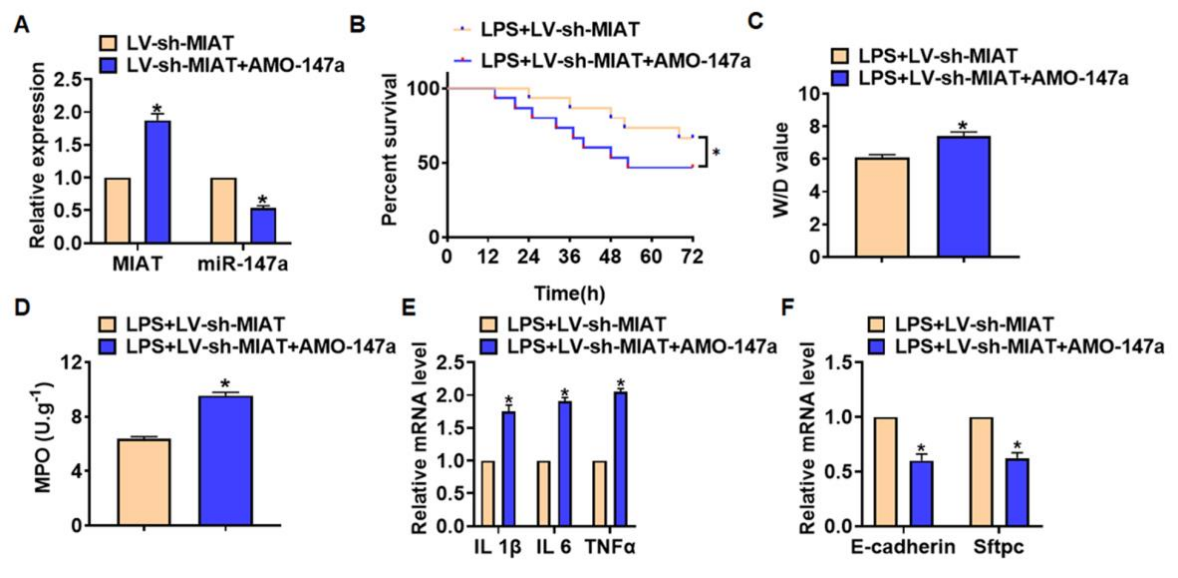
**Deletion of MIAT alleviates LPS-induced inflammation and injury via regulating miR-147a in lung tissues.** LV-sh-MIAT was intratracheally injected into mice LV-sh-MIAT or LV-sh-NC was intratracheally injected into mice. (**A**) The efficiency of sh-MIAT and AMO-147a was determined by qRT-PCR. (**B**) Survival plots for mice in different groups. (**C**) Wet dry mass ratio (W/D) of lungs was calculated. (**D**) Myeloperoxidase (MPO) of lungs was examined. (**E**) qRT-PCR analysis for IL 1β, IL 6 and TNFα expression. (**F**) qRT-PCR analysis for E-cadherin and Sftpc expression. Data are mean ± SD; *P < 0.05. All experiments were repeated three times.

We then inhibited expression of MIAT with AMO-147a or NKAP in TC1 cells (Figure 6A). Knockdown of MIAT inhibited LPS-induced apoptosis, inflammation response and NF-κB signaling activation (Figure 6B–6G). However, AMO-147a or NKAP removed the beneficial role of si-MIAT on TC-1 cells (Figure 6B–6H). Moreover, LV-sh-MIAT was intratracheally injected into mice with or without AMO-147a, and the expression of MIAT and miR-147a in lung tissues was detected using qRT-PCR (Figure 7A). Followed functional experiments showed that AMO-147a reversed the protected effects of sh-MIAT in LPS-treated lungs (Figure 7B–7F).

## DISCUSSION

Pneumonia is a common infectious disease in children [23]. The pathogenesis of pneumonia is complicated, which is often accompanied by immune response and inflammation, which will seriously damage the function of lung tissue [24]. LncRNA is one of the most important nucleotides in recent years. More and more studies have revealed that lncRNAs can regulate the transcriptional expression of target genes at different stages, and levels of cell growth [25]. Present study showed a high expression of lncRNA MIAT in LPS-induced inflammation and injury of lung tissues and cells. Silencing of MIAT removed the adverse effects of LPS on lung tissues and cells, and inhibited apoptosis level, accumulation of inflammatory factors and activation of NF-κB signaling. Mechanismly, deletion of MIAT protected against LPS-induced inflammation and injury via miR-147a/NKAP axis.

Lung injury is the most direct consequence of pneumonia, which is characterized by inflammatory damage to the alveolar capillary membrane and the production of inflammatory cytokines [26, 27]. Inflammatory response and inflammatory cytokines play an important role in the pathogenesis of pneumonia [28]. Animal experiments have shown that LPS stimulation can induce acute inflammatory response and lead to early histopathological changes in the lung [29]. Inhibition of LPS-induced inflammation may be an effective method for the treatment of pneumonia [30]. We injected LPS into the abdominal cavity of mice to induce pulmonary inflammation. The expression of lncRNA was analyzed by high-throughput sequencing, and the differentially expressed lncRNA was found. Although the differential expression of these lncRNA is affected by LPS stimulation, whether they can regulate the inflammatory response induced by LPS still needs to be further verified, but this method can help us to find possible target molecules. Our data showed a significant increase of MIAT in mice with LPS injection.

As a non-coding RNA, lncRNA can interact directly with DNA, RNA, proteins and play a variety of regulatory functions [31]. MALAT1, CCL2, MALAT1, and HAGLROS, which regulate inflammatory response through inflammation-related mediators, are all involved in the regulation of inflammatory response in lung injury [32–35]. LncRNA MIAT is first identified in heart tissues and known as myocardial infarction-associated transcript [36]. Then followed studies show that MIAT is associate with inflammation of different tissues [37, 38]. LncRNA MIAT was increased in diabetic cardiomyopathy. And the high expression of MIAT is closely related to the production proinflammatory IL-17. Further data indicated that MIAT specific suppressed miR-214-3p expression, then removed the inhibitory effect of miR-214-3p on IL-17 expression [21]. Meanwhile, LPS-induced septic cardiomyopathy elevated MIAT expression in heart tissues. Knockdown of MIAT inhibited the inflammatory response and oxidative stress induced by LPS. Further data showed MIAT could activate RAF6/NF-κB signaling and then accelerate inflammation [39].

In present study, we injected lentiviral plasmid to inhibited MIAT expression in LPS injected mice. Functional data showed that sh-MIAT promoted mouse survival, inhibited LPS-induced inflammation response and protected lung function. Alveolar epithelial cells are important cells in the biological function of lung tissue. And we silenced MIAT expression in mouse lung epithelial cells TC-1. Consistent with *in vivo* results, si-MIAT removed the injury of LPS on TC-1 cells, and inhibited accumulation of inflammatory factors. The inflammatory pathway mediated by NF-κB, STAT3 and other related transcription factors is the main pathway of macrophage inflammatory response [40]. Phosphorylation of NF-κB and other transcription factors into the nucleus can affect the transcription of inflammatory cytokines IL-1β and TNFα in the nucleus [41]. LncRNAs have been proved to play a regulatory role by affecting transcription factors [42]. Therefore, we speculate that, lncRNA MIAT may inhibit inflammation by affecting transcription factors associated with inflammatory pathways. As well, si-MIAT inhibited the phosphorylation of IκBα and p65, thus inhibited the activation of NF-κB pathway.

Furthermore, we found that MIAT acted as a sponge of miR-147a, and miR-147a directly targeted NKAP. Accumulating evidences have shown that lncRNAs regulate miRNA abundance by binding or chelating miRNA, and play the "sponge" role of lncRNAs, thus regulating a series of pathophysiological processes [43]. miR-147a has been reported to relate to non-small-cell lung cancer [44]. And NKAP is a nuclear protein that activates NF-κB [45]. LPS treatment induced the expression of NKAP in human corneal fibroblasts [46]. And present results showed that AMO-147a or NKAP remitted the beneficial effects of si-MIAT on LPS-induced inflammation response. Together, silencing of MIAT alleviates LPS-induced pulmonary inflammation and injury via regulating miR-147a/NKAP axis.

## CONCLUSION

In summary, our data revealed that knockdown of MIAT protected against LPS-induced pulmonary inflammation and injury, which was mediated by miR-147a/NKAP axis. And this study might provide new understanding for pneumonia mechanism, and be helpful for pneumonia treatment.

## MATERIALS AND METHODS

### Establishment of mouse model of pneumonia by LPS

After intraperitoneal injection of 22 mg/mL pentobarbital sodium (diluted with normal saline), the caudal root, hindlimb, and eyelash reflexes disappeared after 10 min, and slow breathing was considered as deep anesthesia. Nasopharyngeal drip: LPS saline solution was prepared according to 167um g/mL. After anesthesia, the mouse head was tilted downward, and the tongue was pulled out with tweezers. The 60 μL LPS saline solution (about 10 μg LPS) was absorbed by a fluid transfer gun and dropped into the oral cavity through the posterior wall of the pharynx. The nostril was quickly pinched and maintained for 30 seconds, and the model was successful when all the liquid was absorbed into the nasal cavity, and slight tracheal rales appeared. 150 μL lentivirus containing MIAT-shRNA/NC-shRNA or AMO-147a was intratracheally injected into mice. 21 days after the establishment of the model, mice were intraperitoneally injected with 3% pentobarbital sodium and were euthanatized by excessive anesthesia with a dose of 90 mL/kg, and the organs and tissues were removed for follow-up study. Moreover, macrophages and neutrophil in alveolar lavage fluid were collected as previous described [47]. The research protocol of this study was approved by the Animal Care and Use Committee of the Jinan People’s Hospital Affiliated to Shandong First Medical University.

### Cell culture and transfection

The TC-1 cell lines (Mouse alveolar epithelial cells) were purchased from the Science Cell Laboratory. Cell lines were cultured in DMEM (Thermo-life, United States) with 10 % FBS (Thermo Fisher, USA) and 100 μL/mL penicillin and streptomycin (Beyotime, China) and placed at 37° C with 5% CO2. The TC-1 cells were plated until the cell density reached 80% confluency of dishes to transfect. Anti-miRNA oligonucleotide of miR-147a (AMO-147a) or small interfering RNA (si-RNA) of MIAT or NKAP and were constructed by Genechem (Shanghai, China). The plasmids transfected with Lipofectamine 2000 (Invitrogen, Carlsbad, CA). LPS was added into cells at a concentration of 100 ng/mL for 6 h.

### MTT assay

TC-1 cells were plated in 96-well plates and we used MTT assay to detect the cell viability. MTT (0.5 mg/mL; Beyotime Biotechnology, China) was added to every well after treatment and incubated for 3 h at 37° C. And 150 μL DMSO was added and incubated for 15 min. We measured the absorbance by Spectrophotometer (Tecan, Austria) at 493 nm.

### qRT-PCR

RNA extraction was performed using trizol reagent. NanoDrop 8000 (Thermo Scientific, Waltham, MA, USA) was used to detect the concentration and purity of RNA. The single-stranded cDNAs were synthesized from 1 μg of RNA. The expression of mRNAs and miRNAs were quantified by RT-PCR with SYBR Green I (Thermo Fisher Scientific, Inc).

### Western blot

After RIPA cleavage, we extracted total protein and measured with BCA method. After quantitative denaturation, protein electrophoresis membrane transfer and blocked. The first incubation and second incubation were carried out according to the operation steps. The expression of the protein was expressed by the gray value. Primary antibodies list: GADPH (ab181602, Abcam), cleaved-caspase3 (ab2302, Abcam), bax (ab32503, Abcam), bcl2 (12789-1-AP, Proteintech), TNFα (17590-1-AP, Proteintech), IL 6 (66146-1-Ig, Proteintech), p-IκBα ((ab92700, Abcam), IκBα (10268-1-AP, Proteintech), p65 (66535-1-Ig, Proteintech) and p-p65 (ab183559, Abcam). The secondary antibodies IRDye700/800 Mouse or Rabbit were produced by LICOR (Lincoln, Nebraska, USA).

### Luciferase assay

HEK293 cells were co-transfected with 20 mmol/L miR-147a mimic or miR-NC together with WT-MIAT/Mut-MIAT or WT-NKAP/Mut-NKAP. Luciferase activity was measured with Dual Luciferase Reporter Assay Kit (Transgene, China) on GloMax20/20 at 48 h after the transfection.

### RIP

We used RIP assay to determine the binding between MIAT/NKAP and miR-147a using Magna RIP™ RNA-Binding Protein Immunoprecipitation Kit (Millipore) as previous study [48]. Briefly, TC-1 cells were transfected with biotinylated miR-147a/miR-NC or NKAP/NC, and the mRNA level of MIAT or miR-147a was detected using qRT-PCR.

### H&E staining

The lung tissues were gathered and fixed in 4% paraformaldehyde for 24 hrs. Then the fixed tissues were embedded in paraffin. Next, Paraffin slicer machine was used to cut slices (5-mm cross-sectional). H&E staining was used to evaluate pulmonary morphology. Lung sections were dewaxed with xylene and treated with ethanol at different concentrations for 5 minutes. Hematoxylin staining for 5 minutes, 5% acetic acid treatment for 1 minute, water rinse. Dye with eosin for 1 minute, rinse with running water. Dehydrate in 70%, 80%, 90%, 100% ethanol for 10 seconds, xylene for 1 minute. Drizzle with neutral gum and seal.

### TUNEL

We used the *in situ* Cell Death Detection Kit (TUNEL fluorescence FITC kit, Roche, Germany) detect apoptotic. We used DAPI to stain nuclei. We used IX73 fluorescence microscope (Olympus, Valley, PA) to analyze fluorescence staining. We used Image-J to count the Total cells and TUNEL positive cells numbers.

### Flow cytometry assay

Cell apoptosis was calculated by Annexin V apoptosis kit (Beyotime, China), and the operating procedure was according to the kit instructions. Briefly, 5×10^5^ cells/mL were centrifuged and resuspended in with Annexin V-FITC and PI solution in darkness for 15 min. Then, Binding Buffer was mixed into the resuspension and detected with instrument. Cell apoptosis level was detected within 1 h.

### Fish

The sample was grown or adhered to or sliced on the cover slide and permeated with 70% ethanol. Hybridization can be done in a traditional laboratory incubator at 37° C within 4 hours. After hybridization, the washing buffer was incubated briefly to remove the excess probe. The total time is 1-1.5 hours. The sample can be imaged using a standard fluorescence microscope.

### Statistical analysis

Data were shown as mean±SD. Student’s t-test or one-way ANOVA was used to compare the groups. P<0.05 was considered significance.
